# Patent foramen ovale and scuba diving: a practical guide for physicians on when to refer for screening

**DOI:** 10.1186/2046-7648-2-10

**Published:** 2013-04-01

**Authors:** Oliver Sykes, James E Clark

**Affiliations:** 1 London Hyperbaric Medicine, Whipp's Cross University Hospital, London E11 1NR, UK; 2 Centre of Human & Aerospace Physiological Sciences, King's College, London SE1 1UL, UK

**Keywords:** Patent foramen ovale, Decompression illness, Arterial gas embolism, Screening

## Abstract

Divers are taught some basic physiology during their training. There is therefore some underlying knowledge and understandable concern in the diving community about the presence of a patent foramen ovale (PFO) as a cause of decompression illness (DCI). There is an agreement that PFO screening should not be done routinely on all divers; however, when to screen selected divers is not clear. We present the basic physiology and current existing guidelines for doctors, advice on the management and identify which groups of divers should be referred for consideration of PFO screening. Venous bubbles after diving and right to left shunts are common, but DCI is rare. Why this is the case is not clear, but the divers look to doctors for guidance on PFO screening and closure; both of which are not without risks. Ideally, we should advise and apply guidelines that are consistent and based on best available evidence. We hope this guideline and flow chart helps address these issues with regard to PFOs and diving.

## Review

### Introduction

**Figure 1:**
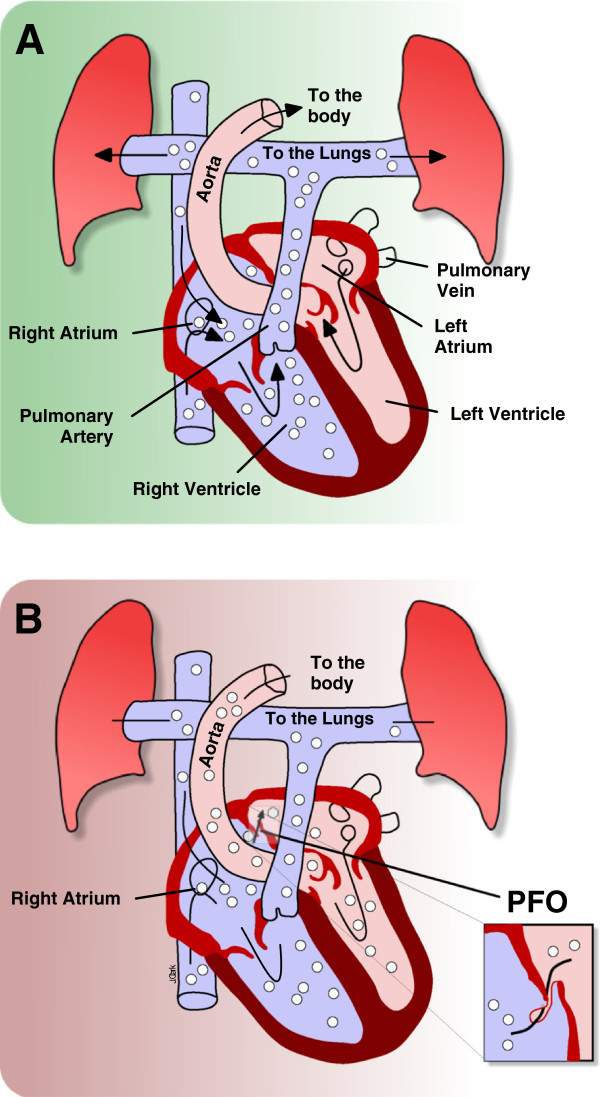
**Paradoxical gas embolism.** Schematic drawing demonstrating the paradoxical gas embolism in a diver with a PFO (**A**); migration of a bubble of gas from the venous system to the left atrium via a PFO, with subsequent systemic embolisation (**B**).

Decompression illness (DCI) encompasses decompression sickness (DCS) and arterial gas embolism (AGE). The differentiation of the pathological processes in practice can be difficult, but the treatment is similar; hence, both are given the modern overarching term of decompression illness. DCS occurs as a result of venous bubbles forming in the tissues and vessels, which can cause mechanical, embolic and biochemical effects with manifestations ranging from trivial to fatal [1]. AGE is caused by arterial bubbles as a result of ruptured lung alveoli from gas trapping in the lungs or blood shunting from the venous right atrial side to the arterial left atrial side of the heart. This is known as a right to left shunt. Symptoms usually appear shortly after, or within 30 min of surfacing, but can have delayed onset. These symptoms are frequently neurological in nature [2-4] and can be profound. The lungs are an effective filter, and a right to left shunt, such as a patent foramen ovale (PFO), is therefore a route for bubbles to avoid this filter and enter the arterial system. This is known as a paradoxical embolism and is depicted in Figure 1. Usually, the blood pressure on the arterial left side is higher than the venous right, which prevents right to left flow. However, this pressure differential is reversed on releasing a Valsalva manoeuvre, causing the right atrium to fill before the left atrium. Unfortunately, 30 to 60 min post-dive is the peak time for bubble liberation [5], which coincides with divers climbing into boats, lifting heavy kit, straining and unconsciously performing Valsalva manoeuvres.

**Figure 2:**
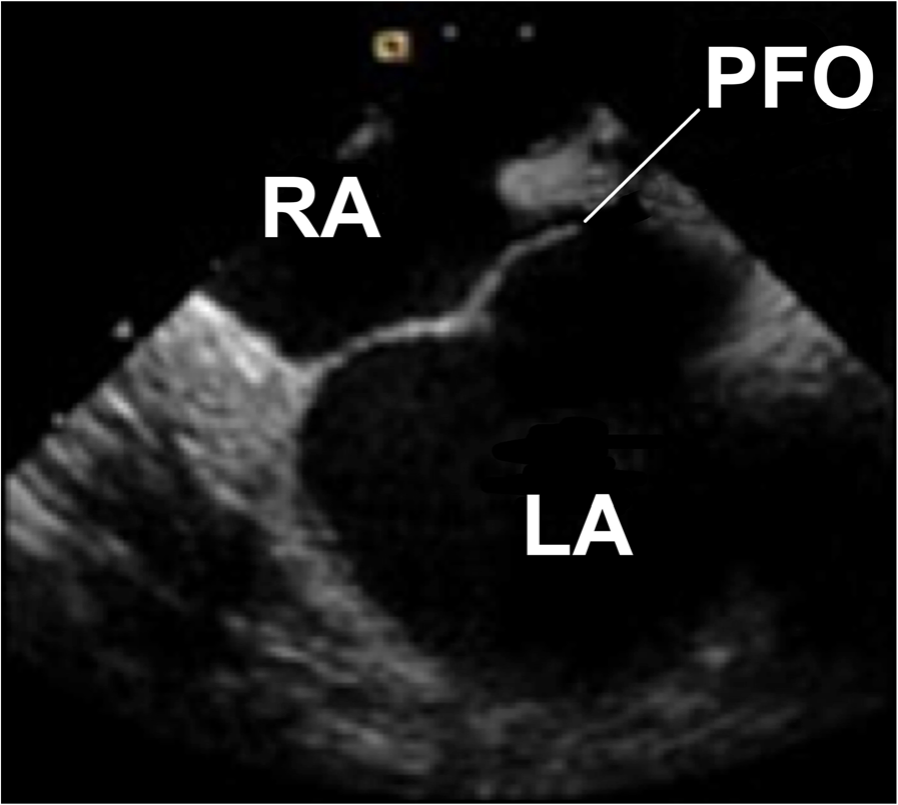
**Intra-cardiac echocardiogram.** Showing the patent foramen ovale, in an adult and in real time, acting as a flap valve between the right atrium (*RA*) and left atrium (*LA*) [6].

In the fetus, the foramen ovale is vital to allow blood to bypass the lungs, which are not in use. On breathing at birth, there is a flap valve effect [6] (Figure 2), and the negative intra-thoracic pressure helps closes this route. While in about 30% there remains a leak, DCI is still a rare event [1,7]. This suggests that not all divers with a PFO are at increased risk of DCI [8]. However, those that are susceptible appear to get the more serious neurological symptoms. Moon examined 91 patients with a two-dimensional echocardiogram, who were evaluated and/or treated for DCS at Duke University Medical Center. Of these 91, 39 had a PFO and 64 of the 91 had more serious symptoms (weakness, dizziness or symptoms of brain abnormalities); 32 of these 64 had a PFO [9] (50%). The risk of DCI by right to left shunting is related to the tissue nitrogen load (i.e. pressure–time profile), the size and characteristics of the shunt and the presence of other factors likely to cause right to left shunting [10,11]. These include occult lung disease, smoking, lung shunts, Valsalva, straining and functional size of the PFO. According to Dr. Peter Wilmshurst, cardiologist of the UK Sports Diving Medical Committee, the requirements for shunt-mediated DCI are a large right to left shunt, a PFO, atrial septal defect or pulmonary arteriovenous lung malformation, a dive profile that liberates venous bubbles profile and also an appropriate inert gas load in critical tissue to amplify embolic bubbles [12].

Divers are taught some basic physiology during their training. There is therefore some underlying knowledge and understandable concern in the popular diving press about the presence of a PFO as a cause of DCI [9,13,14]. Unfortunately, DCI can occur after any dive, even within the depths and time limits of tables and computers, and after the diver has made many hundreds of dives without incident. All divers experiencing problems after diving should consult a diving physician, to whom this guideline is aimed. A list of contact details can be found at http://www.uksdmc.co.uk. Even when performing dives which are inside acceptable and safe decompression algorithms, venous bubbles are very common [2,15,16], and the Divers Alert Network states that:

While 20–30 percent of divers might be expected to have a PFO, decompression illness (DCI) in recreational divers occurs after only 0.005-0.08 percent of dives, clearly much lower than the one in five or six that might be expected if every diver with a PFO and venous bubbles developed DCI. Based on current experience, the estimated risk of a DCI incident characteristic of those correlated with PFO is between 0.002-0.03 percent of dives [17].

Therefore, routine screening of all divers for a PFO is not warranted primarily because the absolute risk of neurological DCI is low and the cost of screening is high [1], and beyond the recommendation not to screen all divers, there are no clear guidelines on when to screen for PFOs in divers who may be at risk of shunt-mediated DCI. Here, we present a practical approach to a common problem of what to do with a diver who may warrant or request a referral for a PFO check. These are guidelines for doctors treating divers and should not be used in place of diver training.

### Current guidelines

According to the UK Sports Diving Medical Committee [18]:

Approximately one quarter of the population have a patent foramen ovale or a small atrial septal defect, but the risk of paradoxical embolism is much greater in those with large shunts [10,19]. Decompression illness is very unusual in sport divers after dives to less than 20 metres and we have not observed neurological decompression illness that appears to be the result of paradoxical embolism in sport divers after dives to that depth. We have observed neurological decompression illness associated with a large shunt in a professional diver who did a working dive at 18 m, which required in-water stops that were performed correctly. It therefore seems reasonable that sport divers known to have intra-cardiac shunts should be allowed to dive shallower than 15 m, provided no other cardiac contra indications exists. If a diver with a shunt wishes to go deeper than 15 m the options include use of nitrox with an air decompression table (to reduce bubble liberation and tissue nitrogen load) and the use of a table such as the DCIEM (Defence and Civil Institute of Environmental Medicine) table which is believed to result in little or no bubble nucleation. It will also be possible for some individuals to return to unrestricted diving after trans-catheter closure of the defect.

For commercial divers, the Health and Safety Executive (HSE) state that [20]:

Examination for the presence of an intra-cardiac shunt is not a requirement for either the initial or the annual examination. However, examination for patent foramen ovale should be performed in a diver who has suffered neurological, cutaneous or cardio-respiratory decompression illness, particularly where there is a history of migraine with aura or where the dive profile was not obviously contributory, since it may contribute to an assessment of the overall risk to the diver of continuing to dive. A positive finding is not necessarily a reason for a finding of unfitness. However, the opinion of a cardiologist with an interest in diving medicine is recommended.

The National Institute of Clinical Excellence (NICE) has produced guidelines on the closure of PFOs in divers, [21] which also emphasises the importance of involving a cardiologist knowledgeable in diving medicine. The assessment of the presence and size of a PFO can be poor and can therefore lead to people getting inappropriate advice and being put at risk. The Undersea and Hyperbaric Medical Society (UHMS) Best Practise Guidelines [22] state that PFO testing may be considered after severe or repetitive neurological DCS and may help in advising divers to modify their dive profiles. Carl Edmond's *Diving Medicine*[23] agrees that the risk from a PFO is not great enough for it to be appropriate to test all divers, and repair of the hole is probably more dangerous than diving with it.

### When to refer

There should probably be different advices for different divers, and we will cover the following categories, based on the current standard operating procedure at London Hyperbaric Medicine: (a) no DCI, (b) one episode of DCI, (c) more than one episode of DCI, (d) migraines and (e) commercial divers.

### No decompression illness

**Figure 3:**
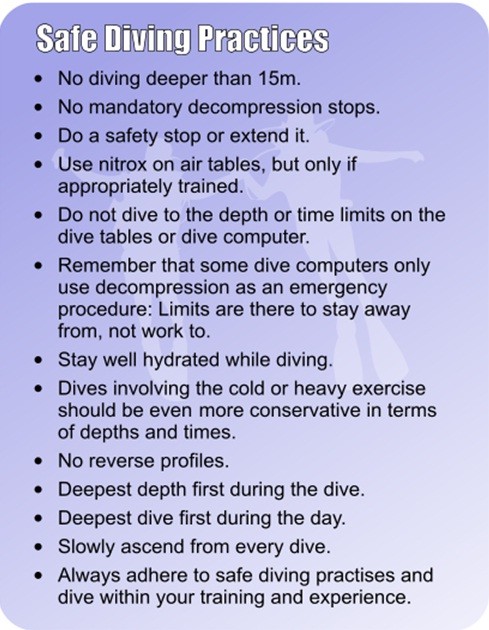
**Safe diving practices.** Courtesy of London Hyperbaric Medicine.

If the diver has not had DCI, discourage the diver from seeking a PFO check. However, consider what the reason for the request might be. Divers often deny symptoms of DCI but worry they may have a PFO. No diving is the only way to guarantee no DCI. Consider also the expense, worry, risk and the possible impact on medical insurance. If concerned about a PFO and the diver wants to continue diving, encourage safe diving practices (Figure 3). A diver's safest profile is a rapid descent to the deepest part of the dive with a gradual ascent, whereas a reverse profile dive is one where the diver spends a prolonged time at the shallowest part of the dive before going deeper for the latter part of the dive. A reverse profile dive maximises nitrogen uptake during the dive and results in a greater risk of bubble formation and, consequently, DCI following ascent. If the diver wants to dive outside these recommendations, then suggest referral to a cardiologist with an interest in diving.

### One episode of DCI

**Figure 4:**
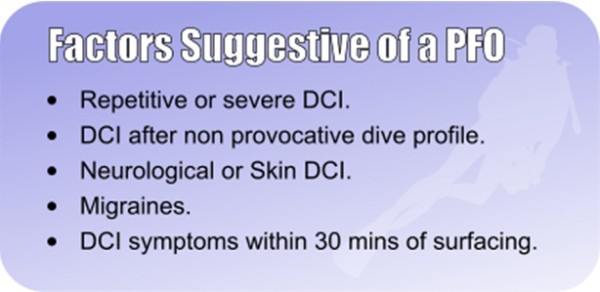
**Factors suggestive of a PFO.** Courtesy of London Hyperbaric Medicine.

**Figure 5:**
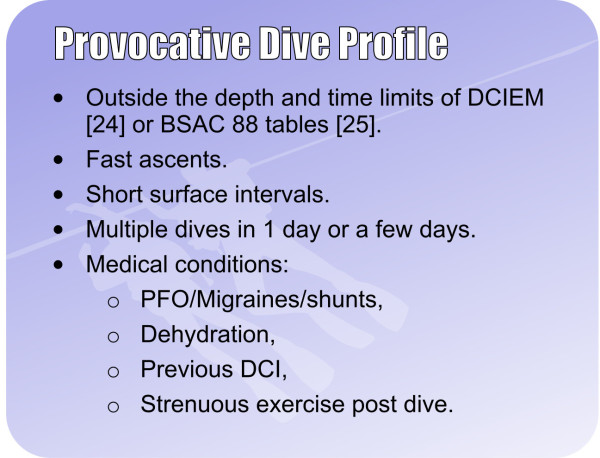
**Provocative dive profile.** Courtesy of London Hyperbaric Medicine.

Discuss whether the diver wants to continue diving, despite being susceptible to DCI. If the diver wants to continue diving, encourage safe diving practices and decide whether there are any factors suggestive of a PFO (Figure 4). If any factors are present, then have a lower threshold for PFO check. If the dive was provocative (Figure 5) and there were no other factors, then encourage safe diving practices and not a PFO check. There is no recommendation to check for a PFO after all types of DCI.

### More than one episode of DCI

As with divers after one episode of DCI, discuss whether the diver wants to continue diving, despite being susceptible to DCI. If the diver wants to continue diving, encourage safe diving practices (Figure 3) and have a lower threshold for screening for a PFO. If the diver clearly understands the risks and agrees to dive to less than 15 m, then no PFO check is necessary. However, the diver may have unrealistic views on what makes a safe dive, and these cases can be difficult. Use the DCIEM [24] or British Sub-Aqua Club 1988 decompression tables [25] to ‘prove’ whether the dive profiles are relatively safe, although DCI can still occur within these tables. A PFO check with a cardiologist with an interest in diving can also be useful in these cases, as this will allow a realistic discussion of the risks of continuing to dive with the diver's cardiac status, as per the UHMS Best Practise Guidelines [22]. We would therefore suggest that a PFO check is discussed with the diver.

### Migraines

Divers with migraine with aura are at increased risk of neurological DCI [26-28]. However, we should encourage safe diving practices (Figure 3) and check whether the medications are appropriate for diving. There is no recommendation to screen for a PFO in divers simply with migraines with aura. However, those with migraines with aura and at least one episode of DCI should probably have a PFO check. Diagnosing migraine with aura is important as migraine without aura and other headaches are not considered a risk factor for DCI or having a PFO.

### Commercial divers

Commercial divers could be defined as those requiring an HSE Commercial Diving Medical for their work. These divers cannot modify their dive profiles and have very clear incentives to continue diving; therefore, stopping diving or encouraging safe diving is not a realistic option. Check whether there are any factors suggestive of a PFO and follow the HSE guidelines above [20].

### Referral, screening and closure

Guidelines for screening for PFOs are difficult to create because the relationship between PFOs and DCI is not clear and also because DCI is rare and most of the tests involve expense, worry and some risk. Cardiac investigations are not always of sufficient quality to pick up all right to left shunts such as pulmonary arteriovenous malformations. There are also a number of ways of testing for a PFO, which may explain why the rates vary. Deciding when to check for and close a PFO can also be difficult but ultimately lies with the cardiologist performing the procedures. PFO checks and closures are done at many centres, but screening and advice on continued diving must come from a cardiologist with an interest in diving.

### The screening procedure

A small dose of bubbles is injected into a large ante-cubital fossa vein, and the diver is asked to perform a Valsalva. Since bubbles show up well on ultrasound, there is then opacification of the right atria and ventricle, and any bubbles that traverse the septum can be easily seen. As far as we know, there have been no reported problems after the dose of intravenous bubbles.

### The closure procedure

**Figure 6:**
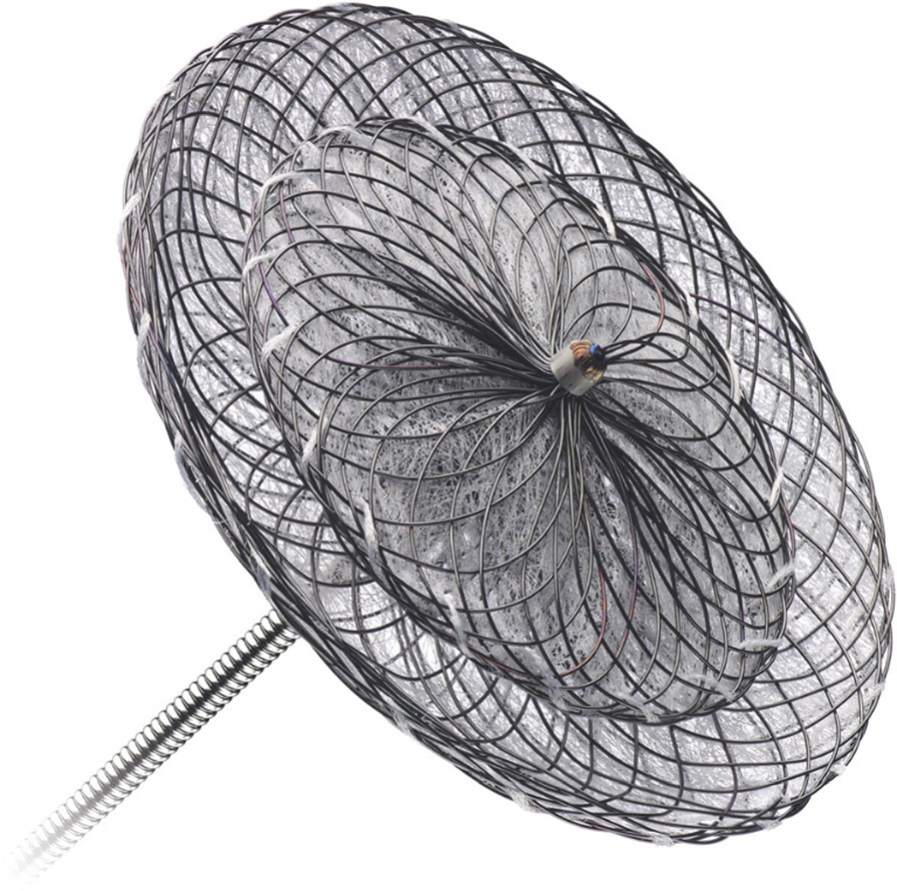
Guidewire and patent foramen ovale occluder device (courtesy of St. Jude Medical).

**Figure 7:**
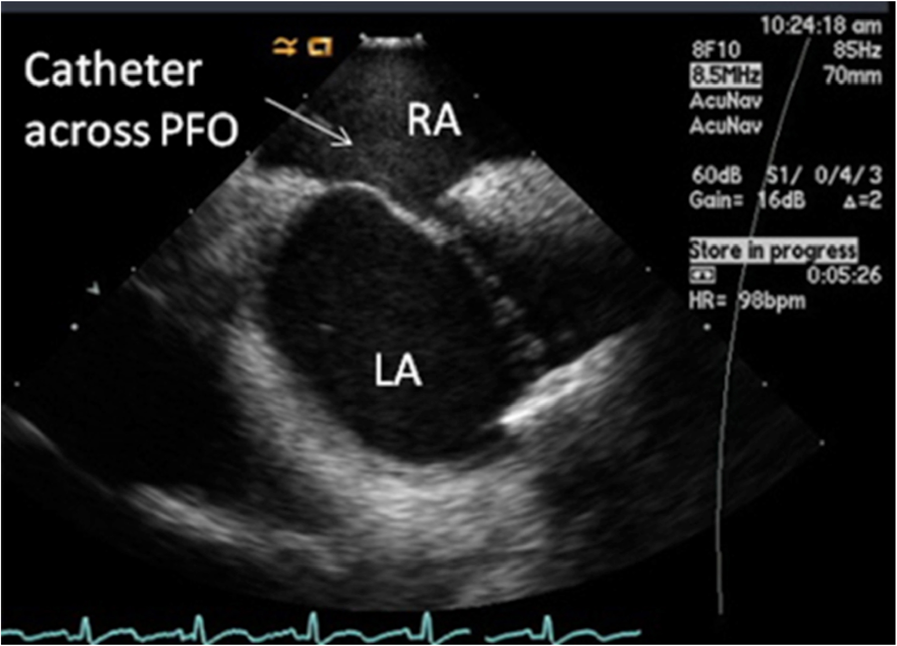
**Intra-cardiac echocardiography.** The guidewire and catheter can be seen in the heart, passing from the right atrium (*RA*), through the patent foramen ovale, into the left atrium (*LA*). The *bright white area* (echo dense) on the wall of the *LA* opposite the *RA* is the occlude device with the guidewire attached [29].

This is performed using a local anaesthetic and sedation, or general anaesthesia, and can be done as a day case. A guidewire and catheter (Figure 6) are inserted through a vein usually in the groin into the heart and through the PFO using imaging guidance [29] (Figure 7). A device is then inserted via the catheter, closing the hole. There is a NICE guidance on *Percutaneous closure of patent foramen ovale for the secondary prevention or recurrent paradoxical embolism in divers* (issued December 2010) [21]. In terms of efficacy and risks, the guidance for patients includes five studies with a total of 1,283 patients who had the procedure for a number of different conditions; the PFO was immediately closed in 1,268 patients (99%) [30] and a further study of 29 divers treated by the procedure for neurological decompression sickness: 23 had returned to diving and experienced no more decompression sickness and 6 were not diving (three as they had only recently had the procedure and three for reasons unrelated to the procedure) [30]. In terms of risks and possible problems, the NICE guidance is useful again [30]:

•In a study of 280 patients, cardiac tamponade was reported in 2 patients (0.71%) who both required further surgery.

•In 2 studies with a total of 992 patients, the device used to close the PFO caused a tear in a large blood vessel of the heart requiring emergency surgery in 1 patient (0.10%). The device fell out and entered the circulation in 7 patients (0.71%).

•Abnormal heart rhythm during or after surgery was reported in 13 of 95 patients (13.68%) in 2 studies of a total of 213 patients.

•As well as looking at these studies, NICE also asked expert advisers for their views who said that in theory, a problem with the heart valves could occur.

It is worth emphasising that the risk post-closure of DCI returns to normal and not zero. The closure also requires checking with repeat echocardiography to ensure closure and a period of antiplatelet therapy which must be completed before returning to diving.

## Conclusions

**Figure 8:**
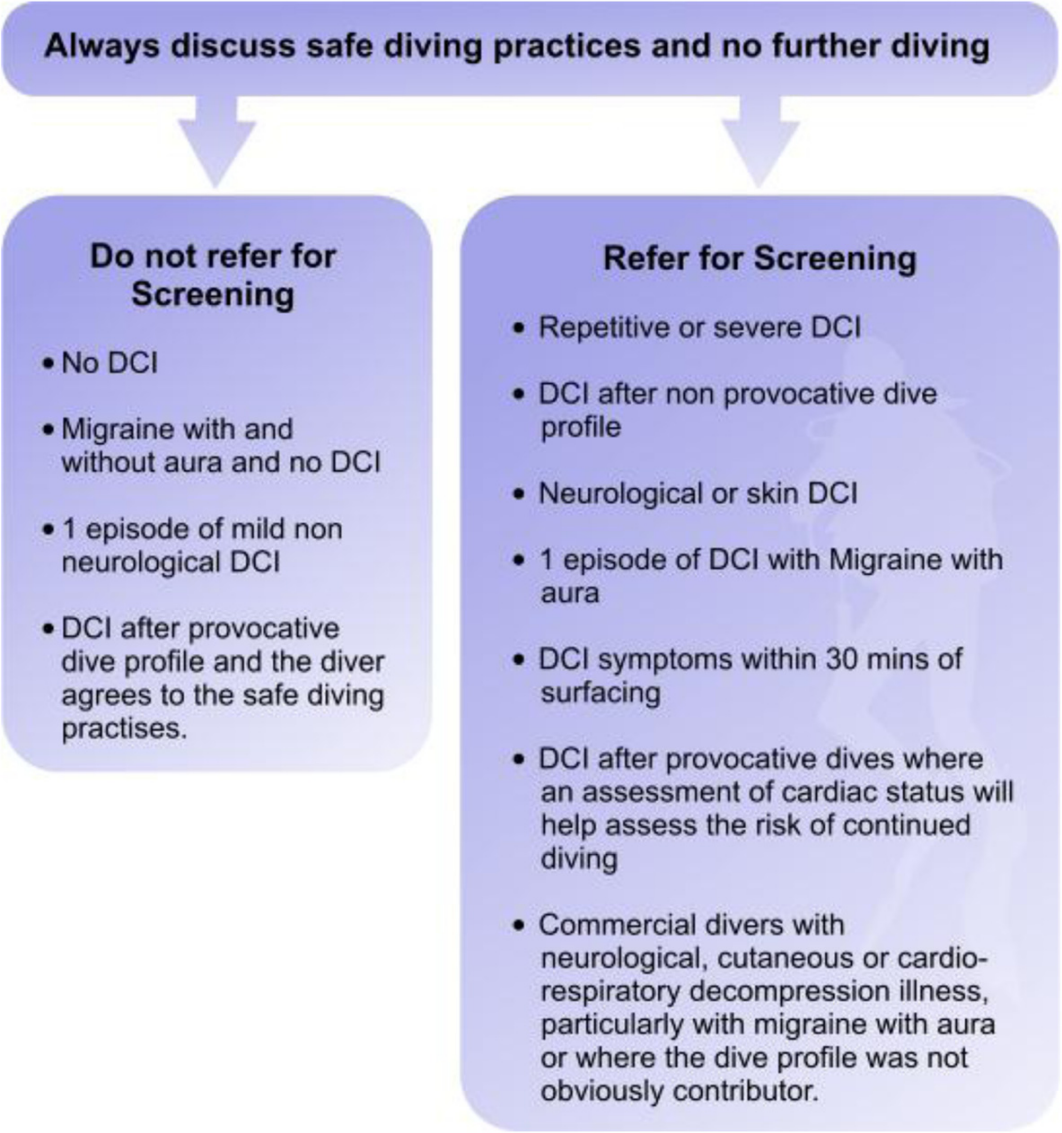
**Flow chart on when to refer for screening by a cardiologist with an interest in diving.** Courtesy of London Hyperbaric Medicine.

Venous bubbles after diving and right to left shunts are common, but DCI is rare. Why this is the case is not clear, but the divers seek doctors' guidance on PFO screening and closure, both of which are not without risks. Ideally, we should advise and apply guidelines that are consistent and based on best available evidence. We hope this guideline and flow chart (Figure 8) help address these issues with regard to PFOs and diving.

## Abbreviations

DCI: Decompression Illness; DCIEM: Defence and Civil Institute of Environmental Medicine; HSE: Health and Safety Executive; NICE: National Institute of Clinical Excellence; PFO: Patent foramen ovale; UHMS: Undersea and Hyperbaric Medical Society.

## Competing interests

JEC is a lecturer in Aerospace and Applied Physiology at King's College, London and runs the B.Sc. Extreme Physiology and M.Sc. in Human Physiology in Extreme Environments and a diving medicine module. OS is paid as a doctor at the hyperbaric unit at Whipp's Cross Hospital and, as part of this work, refers divers for PFO checks.

## Authors' contributions

Both authors (JEC and OS) have made substantive intellectual contributions in conceiving, designing, interpreting, drafting and revising the manuscript critically for important intellectual content and have given final approval of the version to be published. Notably, OS conceived the idea and provided the guideline at London Hyperbaric Medicine. JEC advised on changes to the guidelines and produced graphics. Both authors read and approved the final manuscript.

## Authors' information

OS is currently a senior registrar in anaesthetics in SW London, a PADI Divemaster and a hyperbaric doctor at Whipp's Cross University Hospital, where there are over 100 cases of DCI every year. Some are referred for PFO screening. The guideline for referral of divers for a PFO check was developed by OS in order to help other doctors at the unit refer appropriate cases. OS also writes regularly for *Sport Diver* and contributes to the discussions on the UK Sport Diving Medical Committee forum, where PFO screening is a common theme. JEC is a lecturer and independent researcher at King's College, London within the Centre for Human Aerospace Physiological Sciences and the Cardiovascular Division, respectively. He teaches on the M.Sc. in Human & Applied Physiology programme and undergraduate physiology courses including Human Physiology in Extreme Environments (MSc) and Extreme Physiology (BSc) in diving medicine. He is a British Sub-Aqua Club advanced diver and instructor.
